# Real-time Assessment of the Bidirectional Relationship Between Affective States and Glucose: Protocol for a 14-Day Observational Study

**DOI:** 10.2196/45104

**Published:** 2023-03-22

**Authors:** Chad D Rethorst, Phrashiah Githinji, Rebecca A Seguin-Fowler, Alexandra L MacMillan Uribe, Jacob Szeszulski, Yue Liao

**Affiliations:** 1 Institute for Advancing Health Through Agriculture Texas A&M AgriLife Research Dallas, TX United States; 2 Institute for Advancing Health Through Agriculture Texas A&M AgriLife Research College Station, TX United States; 3 Department of Kinesiology University of Texas at Arlington Arlington, TX United States

**Keywords:** affect, glucose, eating behavior, glucose variability, glucose excursion, ecological momentary assessment, continuous glucose monitoring, continuous glucose monitor

## Abstract

**Background:**

Glucose variability increases cardiometabolic disease risk. While many factors can influence glucose levels, postprandial glucose response is the primary driver of glucose variability. Furthermore, affect may directly and indirectly impact glucose variability through its effect on eating behavior. Continuous glucose monitors (CGMs) facilitate the real-time evaluation of blood glucose, and ecological momentary assessment (EMA) can be used to assess affect in real time. Together, data collected from these sources provide the opportunity to further understand the role of affect in glucose levels.

**Objective:**

This paper presents the protocol for a study that aims to (1) evaluate the feasibility and acceptability of using CGMs along with EMA in nondiabetic populations and (2) examine the bidirectional relationship between affect and glucose in nondiabetic adults with overweight or obesity using a CGM and EMA.

**Methods:**

Eligibility criteria for the study include participants (1) aged 18 to 65 years old, (2) with a BMI of ≥25 kg/m^2^, (3) who are able to read and write in English, and (4) who own a smartphone. Individuals will be excluded if they (1) have type 1 or 2 diabetes or have any other condition that requires glucose monitoring, (2) are pregnant, (3) use any medications that have the potential to alter blood glucose levels or interfere with the glucose sensing process, or (4) have a diagnosed gastrointestinal condition or eating disorder. In a 14-day observational study, participants will wear a FreeStyle Libre Pro CGM sensor (Abbott) and will receive mobile phone–based EMA prompts 6 times per day (randomly within six 2-hour windows between 8 AM and 8 PM) to assess positive and negative affect. Participants will also wear a Fitbit Inspire 2 (Fitbit) to continuously monitor physical activity and sleep, which will be included as covariates in the analysis. Multilevel linear regression models will be used to evaluate the acute relationship between glucose level and affect.

**Results:**

Recruitment started in October 2022 and is expected to be completed in March 2023. We will aim to recruit 100 participants. As of December 12, 2022, a total of 39 participants have been enrolled.

**Conclusions:**

The results of this study will further elucidate the role of affect in glucose variability. By identifying affective states that may lead to glucose excursions, our findings could inform just-in-time behavioral interventions by indicating opportunities for intervention delivery.

**International Registered Report Identifier (IRRID):**

DERR1-10.2196/45104

## Introduction

Effective intervention strategies are required to reduce the immense burden of cardiometabolic disease. Standard behavioral interventions to reduce cardiometabolic disease risk are typically based on population averages [1,2]; however, variability in response to dietary interventions suggests the need for personalized approaches [3]. Precision approaches aim to tailor interventions to an individual’s unique characteristics and contexts [4,5]. For example, just-in-time adaptive interventions aim to facilitate behavior change by delivering an intervention at times and in contexts when individuals are more receptive to behavior change [6]. Identifying opportunities for change requires an understanding of the time-varying factors that influence behaviors.

The potential to measure time-varying factors has increased with the availability of wearable sensors. The use of continuous glucose monitors (CGMs) has resulted in the identification of glucose variability, that is, intraday fluctuations in blood glucose, as a risk factor for the development of cardiometabolic disease [7]. Greater glucose variability predicts increases in glycated hemoglobin (HbA_1c_) in both diabetic and nondiabetic populations [8,9]. Among persons with type 2 diabetes who maintain good glycemic control (HbA_1c_≤7.0%), greater glucose variability was an independent risk factor for coronary artery disease during a 10-year follow-up period [10]. Though evidence in nondiabetic populations is sparse, mechanistically, it has been proposed that glucose variability increases cardiovascular disease risk through increased oxidative stress and inflammation [11], an effect that has been observed among individuals with normal insulin function [12].

While several factors influence glucose levels (eg, stress, physical activity, etc), glucose variability is primarily driven by increases in postprandial glucose [13]. Controlled laboratory studies have demonstrated that several dietary factors impact postprandial glucose, such as meal composition [14,15], meal timing [16], and meal frequency [17]. In real-world settings, eating behavior, and thus glucose variability, is influenced by several individual-level factors. One example of an individual factor that influences eating behavior is affect [18-21]. The relationship between eating behavior and affect appears to be bidirectional as fatigue, stress, and negative mood are associated with increased cravings and food consumption [19-22], while variations in glucose in response to eating may relate to affective and physical feeling states [23-26]. However, research in this area has primarily relied on dietary assessment recall methodologies, which may be prone to bias and recall error [27]. Pairing data from CGMs with data on affect using ecological momentary assessment (EMA), which involves repeated sampling of affect in real time and in an individual’s natural environment, improves the validity of responses [28] and dramatically decreases the time between measurement of mood and glucose.

Combining CGM and EMA data to investigate the relationship between affective states and glucose variability presents an opportunity to support the development of precision interventions to prevent chronic disease by identifying situations that are likely to lead to large glucose increases (ie, an opportunity for change). A recent review [29] of mood and glucose variability in type 1 and 2 diabetes identified only 1 study that used CGMs and EMA to evaluate mood and glucose [25] and called for more research evaluating the relationships between mood or affect and glucose variability. To date, only a small (n=15) pilot study has combined EMA responses with CGM data to evaluate its association with affective and physical feeling states in nondiabetic samples [18]. Thus, the aims of this study are to (1) evaluate the feasibility of conducting a study using CGMs and EMA and (2) examine the bidirectional relationship between affect and glucose in adults using CGM and EMA data.

## Methods

This is a 14-day observational study. Enrolled participants (N=100) will wear a CGM and receive EMA prompts to assess affect 6 times per day over the 14-day period (detailed below).

### Ethics Approval

The study protocol was approved by the Texas A&M University Institutional Review Board (IRB2021-1412F).

### Participants

Participants will be recruited through online social media advertisements, flyers in community settings, and emails to community members who have indicated previous interest in research participation. Interested individuals will complete a brief web-based screening questionnaire to determine study eligibility. Inclusion and exclusion criteria for the study are outlined in Textbox 1.

**Inclusion and exclusion criteria for study participation.**

**Inclusion criteria:**

- Aged 18 to 65 years old
- Have a BMI of ≥25 kg/m^2^
- Able to read and write in English
- Own a smartphone

**Exclusion criteria:**

- Diagnosed with type 1 or type 2 diabetes or any other condition that requires glucose monitoring
- Pregnant women
- Use of any medications that have the potential to alter blood glucose levels or interfere with the glucose sensing process (eg, corticosteroids, metformin, ascorbic acid, aspirin, acetaminophen, etc)
- Diagnosed with a gastrointestinal condition
- Diagnosed with an eating disorder

### Baseline Visit

Upon confirmation of eligibility, participants will attend a baseline visit. Prior to completing any study-related activities, participants will sign an institutional review board–approved consent form. Participants will complete questionnaires to collect demographic data (age, sex, gender, race, ethnicity, and zip code), and study staff will measure participants’ height and weight. Height will be measured with a Seca 213 stadiometer (Seca GmbH). Two measurements will be taken and recorded in centimeters. If a discrepancy of greater than 0.5 cm is noted, additional measurements will be taken until 2 consecutive measurements within 0.5 cm occur. An average of those 2 measures will be recorded for height. Similarly, weight will be measured using a Seca Clara 803 scale, recorded in kilograms, and taken until 2 consecutive measures within 0.5 kg occurs.

#### Glucose Monitoring

Participants will wear a FreeStyle Libre Pro CGM sensor (Abbott) on the upper arm of the participant’s choosing for 14 days. The FreeStyle Libre Pro collects blinded interstitial glucose levels at 15-minute increments.

#### Affect

During the 14-day data collection period, participants will receive EMA prompts delivered to their smartphone via the Ethica mobile app (Ethica Data). Prompts will be delivered 6 times per day. One prompt will be delivered at a random time within each 2-hour window between 8 AM and 8 PM (8 AM-10 AM, 10 AM-12 PM, 12 PM-2 PM, 2 PM-4 PM, 4 PM-6 PM, and 6 PM-8 PM). Each prompt will consist of 10 Likert-scale items that will assess both positive and negative affect, with responses ranging from 1 (not at all) to 4 (extremely): “How (happy, cheerful, relaxed, stressed, anxious, angry, sad, energetic, tired, lonely) do you feel?” The survey will also include 2 “yes” or “no” questions: “Are you (bored, hungry) right now?”

The order of the 10 items will be randomized at each time point. Assessment questions have been validated for their use in daily or momentary assessment [30-32].

### Covariates

#### Physical Activity and Sleep

Each participant will be provided a Fitbit Inspire 2 (Fitbit) to track physical activity and sleep over the 14-day data collection period. In the primary analyses, these variables will be included as covariates, but collection of this data will also facilitate secondary analyses of the complex interaction effects of physical activity, sleep, and affect on glucose level. Data from the Fitbit devices will be processed using Fitabase (Small Steps LLC).

#### Survey

A baseline survey will collect participants’ demographic information and the following covariates:

- Depression symptoms; Patient Health Questionnaire-9 (PHQ-9): the 9-item PHQ-9 is designed to measure depressive symptoms [33].
- Anxiety symptoms; Generalized Anxiety Disorder-7 (GAD-7): the 7-item GAD-7 is a brief scale to identify probable cases of generalized anxiety disorder and to assess symptom severity [34].
- UCLA (University of California, Los Angeles) Loneliness Scale: this is a 20-item scale designed to measure one’s subjective feelings of loneliness as well as feelings of social isolation with established validity and reliability [35].
- Self-Compassion Scale–Short Form (SCS-SF): the 12-item SCS-SF is a short version of the original Self-Compassion Scale [36].
- Coping Strategy Indicators (CSI): the 33-item CSI is a self-report measure of situational coping encompassing the strategies of avoidance, problem-solving, and seeking social support [37].
- Salzburg Stress Eating Scale (SSES): the 10-item SSES questionnaire measures eating in response to stress [38].
- Weight Efficacy Lifestyle Questionnaire–Short Form (WEL-SF): the 8-item WEL-SF measures patients’ confidence in their ability to control eating behavior in challenging situations and was developed as a brief measure for use in research and clinical practice [39].
- Food Cravings Questionnaire-Trait-reduced (FCQ-T-r): the 15-item FCQ-T-r questionnaire measures the frequency and intensity of food craving experiences [40].
- Experience in Close Relationship Scale (ECR-S): the 12-item ECR-S questionnaire concerns romantic relationships [41].

### Follow-up Visit

Upon completion of the 14-day study, each participant will be asked to complete a follow-up visit. Poststudy weight will be measured at the visit (to potentially control for weight change in statistical analysis), and participants will complete a questionnaire to assess their experience during the study. The questionnaire will include both Likert-scale and open-ended questions aimed at identifying any barriers to device compliance. Participants will return the Fitbit and CGM sensor at this follow-up visit.

### Analysis

We will determine compliance to CGM and Fitbit wear (% of wear time) and response rates to EMA prompts (% of prompts responded to). Predetermined compliance values (CGM and Fitbit wear ≥80%) and EMA response rates (75%) will be used to assess the feasibility of the study procedures.

Multilevel linear regression models, which adjust the standard errors for the clustering of EMA and glucose data within individuals and partition the variance of between- and within-person effects, will be used to evaluate the acute relationship between glucose level and affect. The following two outcome variables will be evaluated: (1) average glucose level in the time period between 2 EMA prompts and (2) occurrence of a glucose excursion (defined as a glucose level greater than 1 SD above the mean 24-hour glucose level). Predictor variables will be 2 separate composite scores for positive affect (average of “happy,” “cheerful,” “relaxed,” and “energetic”) and negative affect (average of “stressed,” “anxious,” “angry,” “sad,” and “tired”). Secondary analyses will be used to predict the interaction effects of physical activity, sleep, and affect on glucose level. Similar models will evaluate positive and negative affect as the outcome variable and average glucose level in the hour prior as the predictor. Covariates in the model will include age, sex, BMI, time of day, day of week, physical activity since the previous EMA prompt, and previous night’s sleep. Additional baseline measures will be evaluated as potential covariates and included in the final model if found to significantly improve model fit. Instances of missing EMA or CGM data will be excluded from the analysis.

## Results

Recruitment started in October 2022. As of December 12, 2022, a total of 39 participants have been enrolled. We expect to enroll 100 participants by a target completion date of March 2023.

## Discussion

This project will generate data to elucidate the relationship between affective states and glucose variability, with an aim of ultimately informing intervention development to reduce glucose variability. We hypothesize that a bidirectional relationship exists by which greater negative affect will predict greater glucose variability, while greater glucose variability will predict greater negative affect.

Technological innovations hold promise for improving approaches to understand the relationship between affective states and glucose variability in real-world settings. CGMs allow for real-time collection of data related to postprandial response, while EMA allows for real-time collection of mood states. Pairing these approaches will facilitate a more valid evaluation of the relationships between affective states and eating behavior, as well as provide opportunities for real-time precision nutrition interventions that consider individuals’ physiological and cognitive states. Precision intervention approaches, such as just-in-time interventions, aim to intervene during moments of opportunity [6]. Compliance data, along with the postparticipation questionnaire, will inform changes to study procedures to address the barriers experienced by participants. Evaluation of the relationship between glucose level and affect will aim to identify situations in which glucose excursions occur (opportunity) and can inform interventions to be delivered during those opportunities (ie, meditation or physical activity to improve affect and dietary suggestions that minimize glucose excursions).

## Appendix

Multimedia Appendix 1 Peer review report by Texas A&M Nutrition & Obesity Research Center (tNORC) Pilot and Feasibility Projects (USA).

## Abbreviations

CGM: continuous glucose monitor
CSI: Coping Strategy Indicators
ECR-S: Experience in Close Relationship Scale
EMA: ecological momentary assessment
FCQ-T-r: Food Cravings Questionnaire-Trait-reduced
GAD-7: Generalized Anxiety Disorder-7
HbA_1c_: glycated hemoglobin
PHQ-9: Patient Health Questionnaire-9
SCS-SF: Self-Compassion Scale–Short Form
SSES: Salzburg Stress Eating Scale
UCLA: University of California, Los Angeles
WEL-SF: Weight Efficacy Lifestyle Questionnaire–Short Form

## Data Availability

The data sets generated during this study will be available from the corresponding author upon reasonable request.
