# Supplementary material for: Real-time Assessment of the Bidirectional Relationship Between Affective States and Glucose: Protocol for a 14-Day Observational Study
Source: JMIR Res Protoc. 2023 Mar 22;12:e45104. doi: 10.2196/45104 (PMC10132050; doi:10.2196/45104)
Supplement: Multimedia Appendix 1 [file resprot_v12i1e45104_app1.pdf]

Subject: tNORC Pilot and Feasibility Projects program
Date: Tuesday, September 14, 2021 at 12:58:21 PM Central Daylight Time
From: Rutkowski, Joseph M
To: Chad Rethorst
CC: Wu, Chaodong, Lee, Chanam

Dr. Rethorst,

Thank you for your submission to the Texas A&M Nutrition & Obesity Research Center (tNORC) Pilot and Feasibility Projects program. Your proposal and work is a testament to the excellent research here at Texas A&M that is driving us toward a submission for NIH NORC designation and support. 13 competitive project proposal were received and reviewed by 2 external (non-Texas A&M) faculty with expertise in Obesity and Nutrition; scores were tallied both raw and normalized to each reviewer's mean. We are pleased to inform you that your proposal was one of the top 4 selected for P&F funding. Congratulation. IF any reviewers provided specific strengths and weaknesses of your proposal (this was not required), they are provided below for your reference.

We will follow up with additional information regarding account setup soon.

We thank you for supporting the tNORC mission and, as colleagues, wish you continued success in your research at Texas A&M as we work toward our goal of NORC recognition. We look forward to your progress on your proposed work.

Best regards,
Chanam Lee
Joseph Rutkowski
Chaodong Wu

	Review Criteria				
APPLICANT	Significance	Innovation	Approach	Investigator/Team	New knowledge, technology, or n that can be used to prevent ob metabolic diseas
Rethorst, C.	1.5	2	2	1	

COMMENTS (IF provided):

Overall impact: This tNORC proposal focuses on investigating the utility of continuous glucose monitoring (CGM) in relation to ecological momentary assessment (EMA) of mood states in humans.

Strengths
- Strong research team
- Novel proposal to evaluate the potential bi-directional relationships between CGM and EMA

Weaknesses
- Estimated costs suggest a full enrollment of 100 individuals through the entire study (84 prompts over 14 days), but it is unclear how non-compliance with prompt responses or

individuals who do not complete the entire 14 day course will be handled
- Will general information about timing of meals be recorded? For example, how will individuals on time-restricted feeding/dietary regimens be addressed?

Joseph M. Rutkowski | Assistant Professor
Department of Medical Physiology, College of Medicine | Texas A&M University
2414 Medical Research Building II | 8447 Riverside Parkway | Bryan, TX 77807
ph: 979.436.0576 | rutkowski@tamu.edu

health.tamu.edu
